# WS22 database, Wigner Sampling and geometry interpolation for configurationally diverse molecular datasets﻿

**DOI:** 10.1038/s41597-023-01998-3

**Published:** 2023-02-15

**Authors:** Max Pinheiro Jr, Shuang Zhang, Pavlo O. Dral, Mario Barbatti

**Affiliations:** 1 Aix Marseille University, CNRS, ICR, Marseille, France; 2 State Key Laboratory of Physical Chemistry of Solid Surfaces, Fujian Provincial Key Laboratory of Theoretical and Computational Chemistry, Department of Chemistry, and College of Chemistry and Chemical Engineering, Xiamen University, Xiamen, China; 3 Institut Universitaire de France, 75231 Paris, France

**Keywords:** Quantum chemistry, Molecular dynamics, Chemical physics

## Abstract

Multidimensional surfaces of quantum chemical properties, such as potential energies and dipole moments, are common targets for machine learning, requiring the development of robust and diverse databases extensively exploring molecular configurational spaces. Here we composed the WS22 database covering several quantum mechanical (QM) properties (including potential energies, forces, dipole moments, polarizabilities, HOMO, and LUMO energies) for ten flexible organic molecules of increasing complexity and with up to 22 atoms. This database consists of 1.18 million equilibrium and non-equilibrium geometries carefully sampled from Wigner distributions centered at different equilibrium conformations (either at the ground or excited electronic states) and further augmented with interpolated structures. The diversity of our datasets is demonstrated by visualizing the geometries distribution with dimensionality reduction as well as via comparison of statistical features of the QM properties with those available in existing datasets. Our sampling targets broader quantum mechanical distribution of the configurational space than provided by commonly used sampling through classical molecular dynamics, upping the challenge for machine learning models.

## Background & Summary

In molecular materials, several physical and chemical processes can be triggered or quenched by simply varying the spatial configuration of the atomic constituents. For example, switching the conformation of the retinal molecule from *cis* to *trans*, a key mechanism responsible for vision, yields substantial differences in the measured absorption spectra^1^. The core concept behind such a dependence between the nuclear degrees of freedom (DOF) and the observed quantum mechanical properties is the potential energy surface (PES)^2^. An accurate determination of the potential energy of a molecular system as a function of the atomic positions is the path to unlocking the access and understanding of a multitude of physicochemical observables such as vibrational spectra and chemical reaction rates^3,4^. A conventionally adopted strategy to explore the PES of molecules or molecular assemblies in an unbiased way beyond the equilibrium region is performing ab initio molecular dynamics simulations (AIMD)^5,6^. However, given the intrinsic high-dimensionality of the PESs (3*N*_*at*_−6 dimensions for a molecule with *N*_*at*_ atoms), the exploration of the vast configurational space in chemical processes—involving isomerization, bond-breaking, or proton transfer—requires a broad sampling of the phase space, long timescale simulations, or both, thereby posing a considerable challenge to computational chemistry research.

The tremendous progress in machine learning (ML) within the quantum chemistry (QC) field^7^ is helping to surpass the computational bottlenecks for efficiently constructing high-quality PES of molecules and materials^8,9^. Since then, a great effort has been undertaken to develop increasingly complex machine learning potentials (MLPs)^9–14^, which are nowadays capable of fitting nonlinear PES of organic molecules within the so-called chemical accuracy (1.0 kcal mol^−1^) or better^14–23^. As a standard protocol to probe their performance, the newly developed MLPs are benchmarked against existing compound databases^14^ spanning the configurational space, the compositional space^24^, or both^25–27^. The MD17 database, for instance, is widely used for benchmarking MLPs across configurational space^28^. It comprises ten independent datasets of small to medium-sized molecules with geometries, potential energies, and atomic forces extracted from AIMD simulations performed at a temperature of *T* = 500 K, using a van der Waals corrected PBE functional. This database was recently revised by tightly converging energies and forces for a randomly selected subset of 100,000 geometries to reduce numerical inaccuracies in the original data^29,30^. Despite the notable improvements in data quality,  the revMD17 database still has limitations in evaluating the accuracy of MLPs concerning the description of quantum effects that require a much broader sampling of the PES in terms of energies and configuration space. This issue has gained increasing attention, and recent databases such as VIB5^31^ and QM-22^32^ are targeting global PESs without holes and much broader energy distribution. However, both VIB5 and QM-22 focus on the energies and forces of relatively small molecules with up to 15 atoms, while the surfaces of other quantum chemical properties, such as dipole moments, are also important targets of ML^33^.

The availability of extensive and high-quality quantum chemical data is of paramount importance to advancing the development and application of ML models. With this in mind, we have developed the WS22 database, which aims at complementing previously published datasets in three main aspects: (i) introduce new datasets for molecules of increasing complexity in terms of chemical composition and accessible conformations; (ii) provide a broad and statistically robust representation of PESs with high numerical precision; (iii) provide an extensive set of QC properties that can be used as a target for many different ML tasks.

**Fig. 1:**
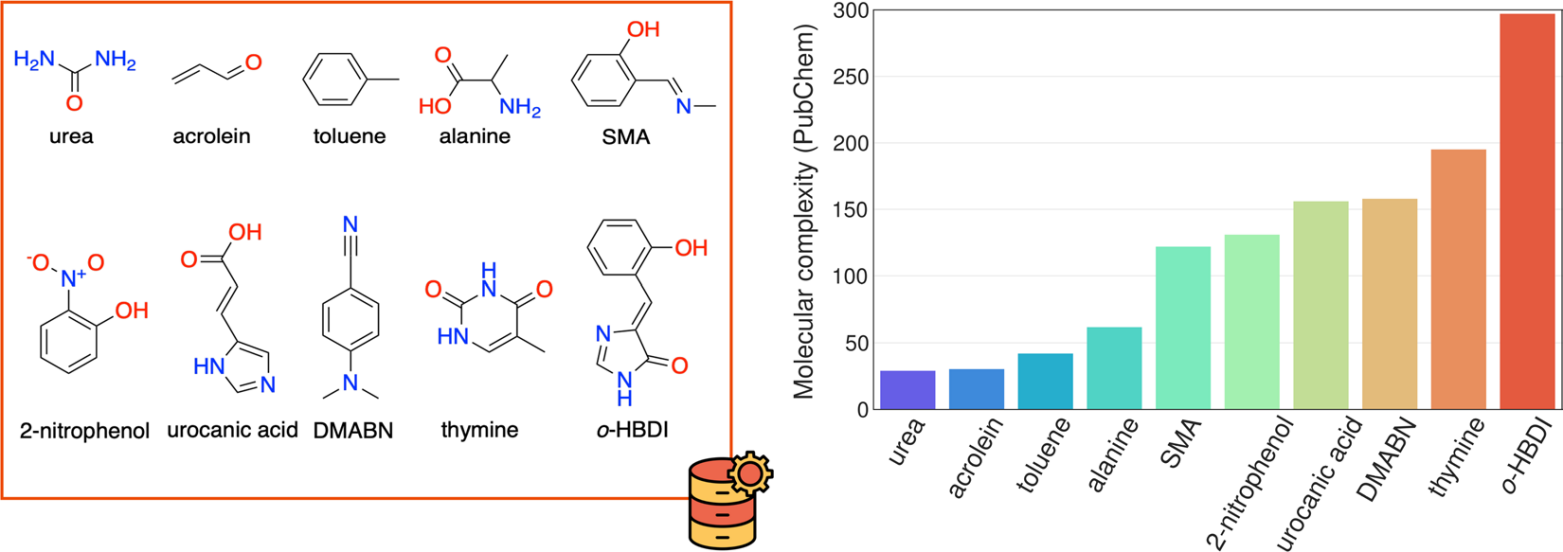
Chemical structures of molecules in the WS22 database (left) and the ranking by their complexity index according to the PubChem website (right). DMABN stands for 4-(dimethylamino)benzonitrile, SMA — 2-(methyliminomethyl)phenol, *o*-HBDI — 4-(2-hydroxybenzylidene)-1,2-dimethyl-1H-imidazol-5(4H)-one.

To accomplish the goal (i), we selected ten molecules of increasing complexity (see Fig. 1 and Table 1), most of which contain flexible functional groups giving rise to different conformations. In fact, some of the molecules in the database are photoactive compounds that can undergo significant structural changes such as *cis*-*trans* isomerization, proton transfer^34,35^, or both^36,37^ when exposed to light. Then, to ensure the configurational diversity required in (ii), we adopted a composing strategy to construct the WS22 database by combining a Wigner sampling approach^38^ with a geometry interpolation scheme^39^. The former method enables us to effectively explore the vibrational degrees of freedom with a dense sampling of non-equilibrium geometries near the local minimum, characterizing different molecular conformations. Complementary, the interpolation scheme allows connecting multiple configurational spaces through a non-linear path that explores internal molecular rotations. A similar strategy has been used to build effective and compact initial training data to perform ML-driven excited-states molecular dynamics^40^. Finally, we accounted for goal (iii) by extending the standard quantities required to fit the PES (i.e., potential energy and forces) to several other chemical properties such as dipole moments, polarizabilities, and HOMO-LUMO energies (Table 2), all computed at a tightly converged DFT level.

**Table 1:** List of chemical compounds used in the WS22 database.

Name	Molecular formula	SMILES	Number of atoms	configuration labels
urea	CH_4_N_2_O	C( = O)(N)N	8	S0, S1
acrolein	C_3_H_4_O	C = CC = O	8	cis, trans
alanine	C_3_H_7_NO_2_	CC(C( = O)O)N	13	M1, M2, M3, M4
toluene	C_7_H_8_	CC1 = CC = CC = C1	15	S0
thymine	C_5_H_6_N_2_O_2_	CC1 = CNC( = O)NC1 = O	15	S0, S1
2-nitrophenol	C_6_H_5_NO_3_	C1 = CC = C(C( = C1)[N + ]( = O)[O-])O	15	S0, S1
urocanic acid	C_6_H_6_N_2_O_2_	C1 = C(NC = N1)C = CC( = O)O	16	cis_1, cis_2, cis_3, cis_4 trans_1, trans_2, trans3, trans_4
SMA	C_8_H_9_NO	CN = CC1 = CC = CC = C1O	19	cis_1, cis_2, trans_1, trans_2
DMABN	C_9_H_10_N_2_	CN(C)C1 = CC = C(C = C1)C#N	21	S0, S1
*o*-HBDI	C_10_H_8_N_2_O_2_	C1 = CC = C(C( = C1)C = C2C( = O)NC = N2)O	22	cis, trans

The last column contains the unique labels that denote the equilibrium reference conformations from which new geometries are sampled using Wigner distribution.

**Table 2:** Description of the data structure used in each molecular dataset to store the quantum chemical properties collected from the Gaussian 09 outputs for the 120,000 geometry configurations.

No.	Quantity	Units	Shape	Description
1	Z		(n_atoms,)	Atomic numbers of nuclei
2	R	Å	(120000, n_atoms, 3)	Cartesian coordinates
3	F	kcal mol^−1^ Å^−1^	(120000, n_atoms, 3)	Atomic forces
4	Q	*e*	(120000, n_atoms, 1)	Mulliken charges
5	D	D	(120000, 3)	Dipole moment
6	P	${a}_{0}^{3}$	(120000, 6)	Isotropic polarizability
7	RC	GHz	(120000, 3)	Rotational constants
8	HL	eV	(120000, 2)	HOMO and LUMO energies
9	E	kcal mol^−1^	(120000, 1)	Potential energy
10	R2	${a}_{0}^{2}$	(120000, 1)	Electronic spatial extent
11	CONF	—	(120000, 1)	Conformation identifier

The quantity column provides the list of acronyms used as keys of the Python dictionary to access the corresponding properties of the dataset. n_atoms is the number of atoms in a molecule.

In total, the WS22 database contains 1.18 million equilibrium and non-equilibrium molecular geometries with associated quantum chemical properties equally distributed over ten independent datasets corresponding to molecules consisting of 8 to 22 atoms. Owing to its configurational diversity and chemical complexity, we believe that the WS22 database will help probe the performance and guide the development of advanced MLPs and other ML models by raising the challenge of the learning task to a higher level.

## Methods

**Fig. 2:**
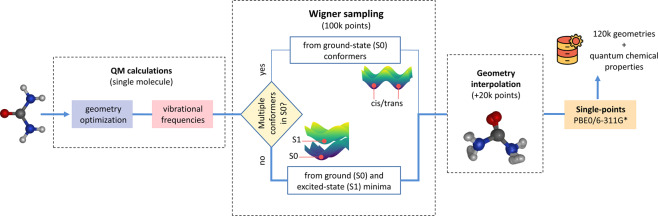
Schematic diagram summarizing the overall workflow employed in the data generation of the WS22 database. The urea dataset is used as an example, with the main steps of the data generation pipeline highlighted with the thicker blue line.

The pipeline for the database construction can be divided into three sequential steps as described in the subsections below. This workflow is also summarized in Fig. 2.

### Geometry optimizations & frequency calculations

As a first step, the equilibrium geometry of each molecule in the database (Fig. 1) is determined either for different conformers or the minima of the two lowest electronic-state surfaces (ground-state, S_0_, and first excited-state, S_1_) depending on the molecule considered to build the dataset. The only exception is toluene, for which no relevant distinct conformations are available, so the equilibrium structure was obtained only for the ground-state surface. This molecule was included in our database for comparison purposes with the MD17 dataset.

All geometry optimizations were performed without symmetry constraints using density functional theory (DFT) with the hybrid density functional PBE0^41^ in conjunction with the 6–311 G* basis set^42^. The Gaussian 09 program^43^ was used to perform all electronic structure calculations in this Data Descriptor. Following the protocol described in ref. ^29^ for the revised MD17, here we used the SCF = VeryTight and Integral(Grid = UltraFine) keywords of Gaussian to tightly converge the electron density and total energy, aiming to achieve a noiseless description of the chemical properties included in our database. To ensure that the final geometries correspond to a (local) minimum in the PES, a tight convergence criterion was also adopted for optimization. The lowest-energy nature of the optimized structures was further confirmed by frequency calculations in which only positive values were found. In the case of excited state calculations, the linear-response time-dependent DFT^44,45^ approach was used for geometry optimizations and frequency calculations with the same theory level, PBE0/6–311 G*. Frequencies are provided in our database, and additional information (zero-point energies as well as internal energies, enthalpies and Gibbs free energies at 298 K) is given in Table 3 for all equilibrium geometries.

**Table 3:** Internal energies, enthalpies, and Gibbs free energies at 298 K as well as zero-point energies in Hartree for equilibrium geometries calculated at PBE0/6–311 G*.

Name	Configuration labels	Zero-point energy	Internal energy	Enthalpy	Gibbs free energy
urea	S0	0.064889	−225.003897	−225.002952	−225.034471
	S1	0.063203	−224.833948	−224.833004	−224.864906
acrolein	cis	0.061739	−191.659894	−191.658950	−191.690666
	trans	0.061651	−191.662575	−191.661631	−191.693219
alanine	M1	0.109337	−323.341211	−323.340266	−323.378521
	M2	0.109246	−323.340162	−323.339218	−323.377569
	M3	0.109374	−323.343497	−323.342553	−323.381201
	M4	0.109444	−323.341246	−323.340302	−323.379244
toluene	S0	0.128413	−271.147757	−271.146813	−271.184937
thymine	S0	0.116300	−453.619459	−453.618515	−453.659639
	S1	0.111405	−453.462545	−453.461601	−453.505670
2-nitrophenol	S0	0.109033	−511.416925	−511.415981	−511.457003
	S1	0.106279	−511.303268	−511.302324	−511.343685
urocanic acid	cis_1	0.122180	−491.640193	−491.639249	−491.681775
	cis_2	0.121977	−491.631662	−491.630717	−491.673906
	cis_3	0.121620	−491.626216	−491.625272	−491.668891
	cis_4	0.121540	−491.622001	−491.621057	−491.665514
	trans_1	0.121364	−491.632694	−491.631750	−491.675827
	trans_2	0.121303	−491.631542	−491.630598	−491.674790
	trans_3	0.121287	−491.631045	−491.630101	−491.674367
	trans_4	0.121156	−491.629269	−491.628325	−491.672802
SMA	cis_1	0.155418	−439.636104	−439.635160	−439.679357
	cis_2	0.154860	−439.633528	−439.632583	−439.678102
	trans_1	0.156101	−439.652596	−439.651652	−439.694504
	trans_2	0.155229	−439.629338	−439.628394	−439.672614
DMABN	S0	0.173349	−457.823813	−457.822869	−457.871344
	S1	0.168365	−457.693323	−457.692379	−457.743172
*o*-HBDI	cis	0.168252	−645.071678	−645.070734	−645.119328
	trans	0.167892	−645.060147	−645.059203	−645.107665

Linear-response time-dependent DFT approach was used for S_1_ minima.

**Fig. 3:**
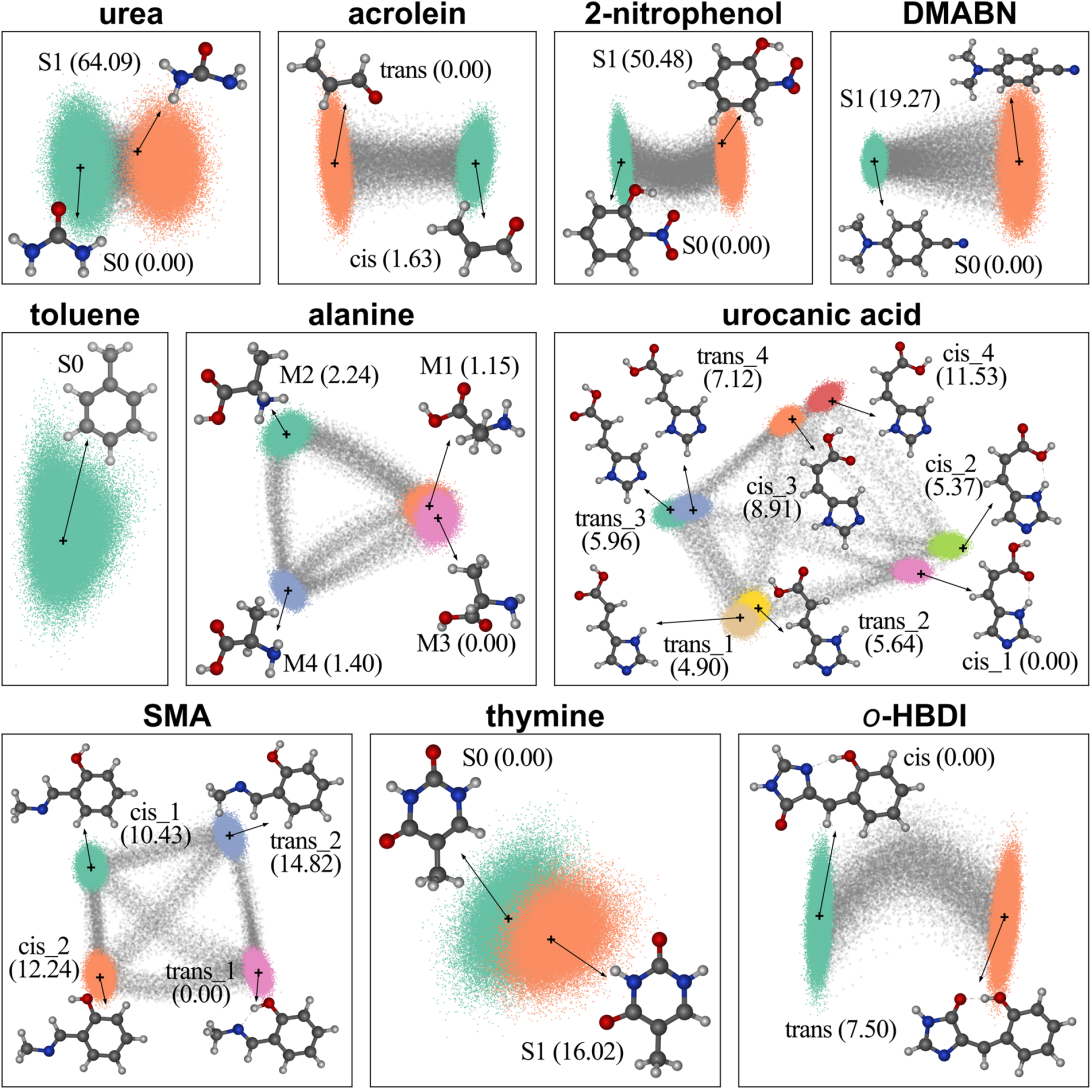
Illustration of the conformational diversity of the WS22 database showing equilibrium geometries of the considered conformers as well as principal component analysis (PCA) of all structures in each molecular dataset. In PCA, the molecular geometries were first converted into a pairwise nucleus-nucleus distance matrix descriptor with only unique off-diagonal elements and then normalized with a min-max scaling to use as input for the PCA projections. The gray markers represent geometries interpolated between pairs of accessible conformations, and the location of equilibrium structures on PCA plots are indicated by ‘+’ markers and arrows. The total energies of each conformer relative to the most stable one are shown in kcal mol^−1^ in parentheses.

For urea, 2-nitrophenol, DMABN (4-(dimethylamino)benzonitrile), and thymine, the geometry optimization calculations were carried out for both S_0_ and S_1_ states. The equilibrium structures of 2-nitrophenol and DMABN in the S_1_ state are highly distorted with a twisting angle of 90° for the nitro and dimethylamino groups, respectively, in relation to the planar ground-state geometry (Fig. 3). In the case of urea, the most significant difference between the S_0_ and S_1_ equilibrium geometries is the pyramidalization of the carbon atom in the excited state conformation. This optimization strategy using the first excited state allows us to sample a much broader region of the configurational space, as will be described in the next subsection.

The remaining five molecules composing our WS22 database (acrolein, alanine, SMA (2-(methyliminomethyl)phenol), urocanic acid, and *o*-HBDI (4-(2-hydroxybenzylidene)-1,2-dimethyl-1H-imidazol-5(4H)-one)) are characterized by multiple conformations accessible in the electronic ground-state. Thus, in this case, we search for the minimum energy structures by performing geometry optimizations starting from different conformers. Four low-lying conformers corresponding to different local minimum structures (M1 to M4) were found for alanine with a maximum energy difference of 2.24 kcal mol^−1^. The molecular geometries of acrolein and *o*-HBDI were optimized for single *cis* and *trans* conformations, which differ in energy by −1.63 kcal mol^−1^ and 7.50 kcal mol^−1^, respectively. Concerning the other two molecules completing the database, several conformations are energetically accessible in the ground state. In the case of the Schiff base SMA, we have selected and optimized the four lowest energy structures reported in ref. ^46^. These structures are named in our work as *cis_i* and *trans_i*, *i* = 1, 2, where the maximum energy difference of 14.82 kcal mol^−1^ is observed between the two *trans* conformers. Finally, urocanic acid is the compound in the database with the largest number of conformers. A total of eight molecular structures, four *cis* and four *trans* isomers were selected from ref. ^47^ to perform the geometry optimizations. The ground state energies of these structures span a range of about 11.53 kcal mol^−1^ in the following energetic order: *cis_1* < *trans_1* < *cis_2* < *trans_2* < *trans_3* < *trans_4* < *cis_3* < *cis_4*. Each of the eight urocanic acid isomers can be inter-converted into the other by rotations of the carboxylic and imidazole groups attached to the central carbon-carbon double bond and cis-trans isomerization around this double bond. All the equilibrium structures are shown in Fig. 3.

### Molecular geometries generation

#### Wigner sampling

A central motivation to build a dataset beyond MD17 is that the quantum mechanical distribution of the configurational space is much broader than the one provided by classical AIMD at 500 K^48^. The zero-point energy stored in each degree of freedom is usually much bigger than the classical thermal energy, meaning that the vibrational amplitudes are much more prominent in the quantum than in the classical picture. A straightforward way of sampling the configurational space (or, more generally, the phase space) to match the zero-point energy requirement is through a quantum-harmonic-oscillator Wigner distribution of the nuclei^38^. The optimized molecular geometries and their corresponding harmonic frequencies described in the previous section form the basis for generating an ensemble of non-equilibrium structures sampled from a Wigner probability distribution function^38,49^. This function maps the nuclear wave function—written as a product of ground-state harmonic oscillator wave functions, one for each normal mode—on the classical phase space^50^. Within this approach, each of the *N*_*F*_ = 3*N*_*at*_−6 normal mode coordinates and momenta are randomly sampled according to the probability distribution function

1 $${P}_{W}\left({\bf{Q,\; P}}\right)=\mathop{\prod }\limits_{i=1}^{{N}_{F}}\frac{1}{\pi \hbar }\exp \left(-\frac{1}{\hbar {\omega }_{i}}\left({\omega }_{i}^{2}{Q}_{i}^{2}+{P}_{i}^{2}\right)\right)$$

where ${Q}_{i}={\mu }_{i}^{1/2}{q}_{i}$ and ${P}_{i}={\mu }_{i}^{-1/2}{p}_{i}$ are the mass-scaled coordinate and momentum for each normal mode *i* with coordinate *q*_*i*_ and momentum *p*_*1*_, reduced mass *μ*_*i*_, and angular frequency ω_*i*_. After the sampling, the normal-mode coordinates and momenta are converted to Cartesian coordinates and momenta.

Instead of a Wigner distribution, we could consider increasing the AIMD temperature to match the zero-point energy. Nevertheless, this approach does not reproduce the vibrational ground-state Wigner distribution for two reasons. First, it is prone to zero-point energy leakage^51^. Second, when a molecule is at the ground vibrational level, the quantum and classical distributions peak at different regions of the phase space [see, e.g., ref. ^52^, Ch.6]. These two effects cause the high-temperature classical distribution to differ significantly from Wigner, as discussed in ref. ^48^.

Using the normal mode coordinates of the PBE0 equilibrium structures as input for the Wigner distribution, we generate a total of 100,000 geometries for each molecule in the WS22 database. These geometries were equally distributed into the different conformers. For example, in the case of molecules having equilibrium geometries in the ground and first excited-state or having only one *cis* and one *trans* isomers, 50000 geometries were sampled from each configurational subspace (S_0_/S_1_ and *cis*/*trans*). Urocanic acid is the molecule with the most fragmented sampling in the database, 12500 geometries per conformation. To parallelize and speed up the sampling process, the configurational subspace of each molecule was further divided into smaller datasets of equal size. For all molecules with two equilibrium geometries, for instance, the 50000 geometries generated for each configurational subspace were sampled from the Wigner distribution in five chunks of 10000 geometries. In this case, a different random seed was used to generate each smaller dataset to guarantee the statistical diversity of the sampled geometries. The broad span of configurations for each molecular dataset can be observed in the clustering structure of the principal component analysis (PCA) projections shown in Fig. 3, which were generated using the unique elements of the nucleus-nucleus distance matrix (off-diagonal lower triangle) as a descriptor.

The Wigner sampling calculations used to build all datasets were performed with the initial conditions program integrated into the Newton-X CS (version 2.2-B08) package^53^.

#### Geometry interpolation

To extend the coverage of the configurational space beyond the vibrational degrees of freedom probed by Wigner sampling, we augmented the datasets by performing a series of geometry interpolations between every possible combination of stable conformers. The method we used for the interpolation acts directly on the Cartesian coordinates space by finding the optimal geodesic curve on a Riemannian manifold with a metric defined by a set of redundant internal coordinate (RIC) functions. In this procedure, the geodesic path (a generalization of a straight line in Euclidean space) between the initial and final geometries is approximated by a piecewise integral evaluated on the RIC metric space. The number of piecewise segments corresponds to the intermediate geometries in the interpolation procedure, which in our case is set to 20. Finally, least-squares minimization is applied to each segment to obtain a smooth geodesic curve between the two input geometries. For detailed mathematical derivations and implementation of the geodesic interpolation method, we refer the reader to the original paper by Zhu *et al*.^39^. As demonstrated in this reference, these geodesic curves capture the topographical structure of the molecular PES, thus providing a good approximation for the true minimum energy path between reactants and products in chemical reactions. In our case, the geodesic paths create a smooth link between two different conformational spaces (e.g., from *cis* to *trans* conformations) by acting mainly on the molecular rotational degrees of freedom. In this way, we included in the datasets molecular structures far from equilibrium, corresponding to regions near transition states on the PESs, which are inaccessible via Wigner sampling.

The data augmentation process used here consists of two steps. First, we randomly picked two molecular geometries from different conformational spaces, corresponding to the endpoint geometries in the geodesic curve search. Then, the Python program provided by ref. ^39^ [https://github.com/virtualzx-nad/geodesic-interpolate] was used to generate 20 interpolated geometries along the geodesic path. This procedure was repeated multiple times for each configurational dataset of WS22 until 20,000 new molecular geometries were sampled. Note that the interpolated geometries are distributed within all the unique pairs of conformation types. For example, in the case of the alanine dataset, we have six possible combinations of conformers where two of them, M1 and M3, correspond to relatively similar structures (see the overlapping clusters in Fig. 3). Thus, for the M1-M3 path, we generated 2000 molecular geometries, while the other five combinations of conformers contributed with 3600 interpolated geometries. This counting of the number of interpolated geometries per conformation pair can be easily done by selecting the specific string identifier in the CONF variable of the dataset, as described in the Data Records section (see Table 2).

### Single-point DFT calculations

Once the molecular geometries have been generated, we moved to the last and most computationally intensive step of our database construction pipeline, which is the single-point (SP) electronic structure calculations. In total, we performed 1.18 million SPs to label (using ML terminology) all molecular geometries in the database with the respective quantum chemical properties. The atomic forces were calculated for every geometry in the datasets via analytic derivatives by requesting the Force keyword in the input of the electronic structure program. These calculations were carried out with the same DFT method and basis set as in the geometry optimizations and frequency calculations, i.e., PBE0/6–311 G*.

## Data Records

**Fig. 4:**
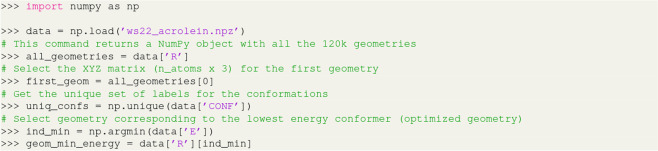
Python code snippet to select specific molecular geometries stored in the database. The code assumes that the NPZ file containing the dataset has been downloaded to a local directory.

All the relevant electronic structure information available in the Gaussian 09 output files are collected and stored in independent NumPy npz format for each compound in the database. This compact binary file has a dictionary-like structure whereby the molecular configurations and the calculated chemical properties can be accessed by querying the dictionary with a string key used as a shortcut for the property’s name. Each query returns a NumPy array object with the number of molecular configurations corresponding to the first dimension of the array. For example, using the key R as the dictionary entry, one can gather information on all molecular geometries stored in the dataset as a NumPy 3D tensor, where the first dimension corresponds to the sample indices and the other two dimensions store the XYZ Cartesian coordinates (in Angstroms) of each sample (see Fig. 4). A full description of the data records with information on the dictionary entries, units, and NumPy array shape is provided in Table 2. The recorded data corresponds to all single-point calculations performed for Wigner sampled geometries and interpolated geometries as well as the minimum energy structures of each conformation obtained via geometry optimization. To access the minimum energy structures in the full dataset, one can search for the index corresponding to the lowest energy value in the E array, as exemplified in the Python code of Fig. 4. For completeness, these minimum energy structures corresponding to the optimized geometries for each conformation are made available in the WS22 database as separate XYZ files per molecule. In addition to the calculated quantities, we also provide an array of strings that tags each geometry with a label corresponding to the conformation identifier (CI) and a dash-separated pair of CI labels in the case of the interpolated geometries. The WS22 database is open access and publicly accessible via ZENODO.ORG data repository^54^, where we also include a README file presenting a summary of the data structure and composition along with a brief technical description on how to access the information stored using Python.

We also provide a zip archive with XYZ geometries and Gaussian 09 output files of frequency calculations for the equilibrium geometries.

## Technical Validation

### Conformational diversity

Our database was designed to cover as wide as possible a range of the conformational degrees of freedom of flexible organic molecules without relying on expensive ab initio MD simulations. To this end, we combined a Wigner sampling approach—from which we obtain a dense grid of geometries spread around the equilibrium regions of the PES(s)—together with a geometry interpolation strategy to construct the transition paths between every pair of conformations. This data augmentation scheme aims to cover a broad PES region composed of geometries distorted along the energy barrier pathways connecting the different minima. The configurational diversity of the datasets generated with our sampling strategy is qualitatively verified by the two-dimensional projection of the geometry space using principal component analysis (PCA), as shown in Fig. 3. In fact, one can see in the PCA diagram the formation of well-separated clusters for most of the molecular datasets, which indicates the successful sampling of largely different regions of the PESs. Moreover, by including interpolated geometries in the dataset (see gray stars in Fig. 3), we ensure that important rotational degrees of freedom non-accessible via Wigner sampling are also covered. These interpolated geometries (20,000 points, corresponding to ~16% of each dataset) create a smooth path connecting different conformational spaces, thus mapping energy barrier regions of the PES that might require long MD simulations to be satisfactorily sampled. This geometry interpolation can also be a helpful data augmentation strategy in MD trajectories to fill configurational gaps, especially to densify steepest regions of the PES where the sampling is statistically less accurate.

**Fig. 5:**
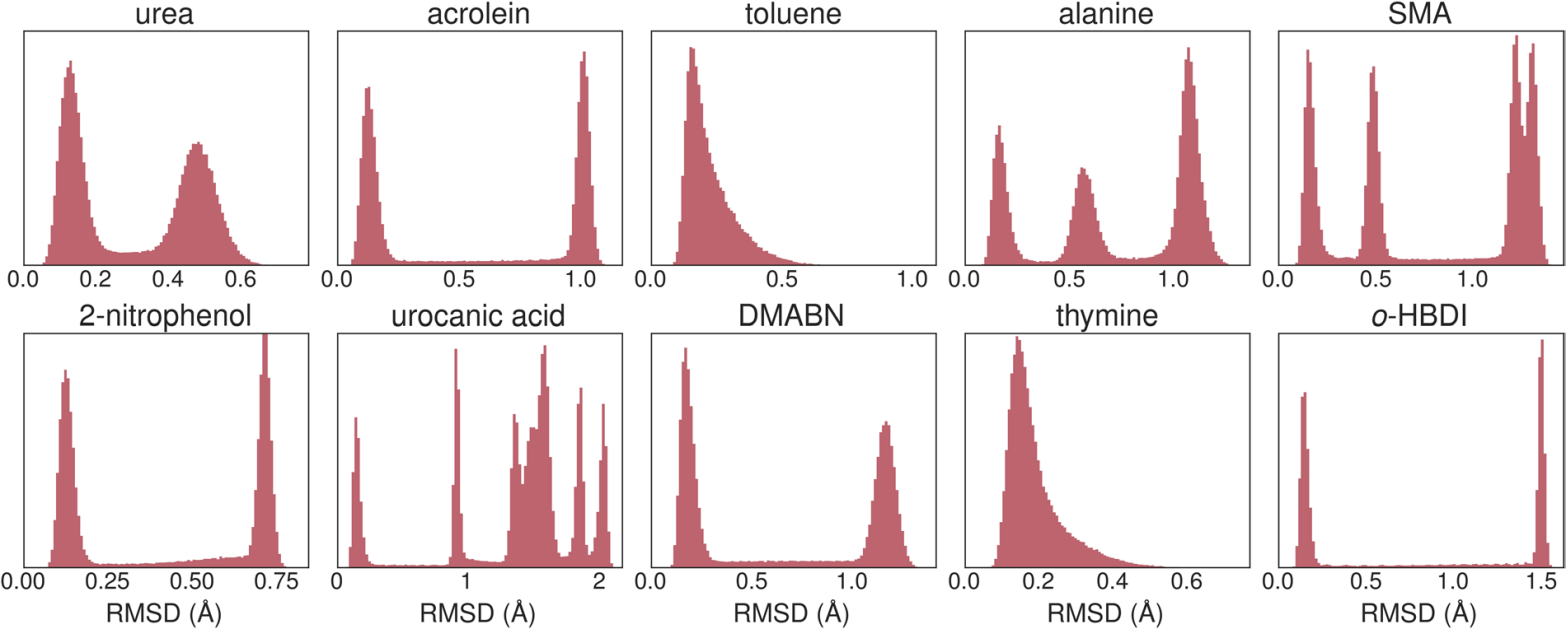
Distribution of geometries deformation with respect to the minimum energy structure as given by the root-mean-squared deviation (RMSD). The Kabsch algorithm was applied to each pair of geometries for the optimum alignment before calculating the RMSD.

To quantitatively assess the conformational diversity in the WS22 dataset, as given by the extent of geometries distortions, we show in Fig. 5 the distribution of root-mean-squared deviation (RMSD) between each sampled geometry and the minimum energy structure. In these calculations, each pair of molecular geometry is pre-aligned by the Kabsch algorithm^55^ to obtain the optimal RMSD. It is visible from the plots in Fig. 5 that the overall geometries’ deformations are widely spread in an RMSD range that can vary from 0.7 Å (urea and thymine) up to 2.0 Å (urocanic acid). The multiple localized and well-separated peaks in the RMSD distribution (except for toluene and thymine) indicate the rather different subspaces of molecular conformations sampled by the Wigner distribution. We also see the crucial role of geometry interpolation in filling the gaps between the different conformational subspaces.

**Fig. 6:**
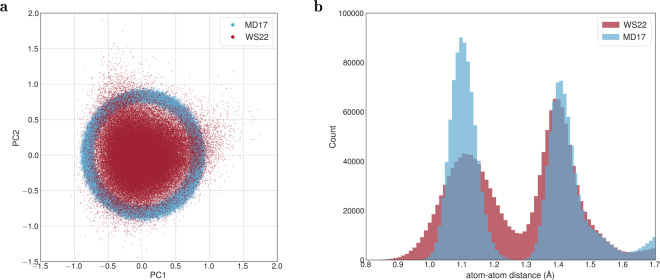
Comparison of geometries space distribution for the MD17 and WS22 toluene datasets. (**a)** Two-dimensional PCA projection of the 100k molecular geometries represented by the normalized pairwise distance matrix descriptor. (**b**) Histogram of the pairwise nucleus-nucleus distances focusing on the typical bond length range.

Now turning attention to toluene, we analyze in Fig. 6 the differences in the conformational space covered by the MD17 and WS22 datasets due to the different sampling approaches. Using the PCA projection again to visualize the geometries’ distribution in a 2D map (Fig. 6a), we can see that the MD17 data points are concentrated on a ring of a relatively small radius. This distribution seems to be a signature of the classical approximation used in the molecular dynamics simulations of MD17. In contrast, the Wigner sampling approach used in WS22 is quantum by construction, and, as such, it generates a very different geometry distribution, having a higher density at the center of the PCA plot and spreading out radially over a large area. Noticeably, in the WS22 dataset, the geometries’ distortion along the two principal components extends beyond the ring area covered by MD17. The differences between the two datasets are reflected in the histogram of pairwise atom-atom distance (Fig. 6b), where we see that WS22 data exhibits broader peaks than the MD17-toluene in the typical bond length range (1.0–1.7 Å). This is also expected to impact the distribution of the typical target quantities for MLPs, potential energy and forces, as we will see in the next section.

### Energy and forces coverage

In addition to the demonstrated structural diversity of our database, we also validated  its broad PES coverage with respect to the potential energy and atomic forces by comparing the present results with the MD17 database. Taking toluene as an example, we can see from Fig. 6 that the energy distribution in the WS22 dataset obtained via Wigner sampling is not only broader than that of MD17 but is also centered on a much higher energy value, 44.8 kcal mol^−1^ and 20.0 kcal mol^−1^, respectively, as given by the median values. The spread in the energy distribution measured by the standard deviation (excluding outliers) is almost three times larger in the WS22 dataset, 12.9 kcal mol^−1^ than in MD17, 4.8 kcal mol^−1^. This difference is a consequence of the quantum effects on the vibrational amplitudes present in the Wigner distribution by construction but absent in the *ab initio* MD simulations used to build the MD17 dataset^48^. Indeed, a key distinction between sampling the nuclear coordinates via quantum distributions and classical dynamics is the amount of energy deposited in each degree of freedom. In the quantum distribution within harmonic approximation at 0 K, this energy is the harmonic zero-point energy. For a normal mode vibrating at 1000 cm^−1^, it amounts to 0.12 eV. On the other hand, in classical dynamics of a canonical system equilibrated at temperature *T*, the equipartition principle ensures that each degree must have *k*_*B*_*T*, which corresponds to 0.04 eV at 500 K (as employed in the MD17 dataset). Thus, nuclear geometries sampled from a quantum distribution have a much broader distribution than when sampled from classical dynamics.

**Fig. 7:**
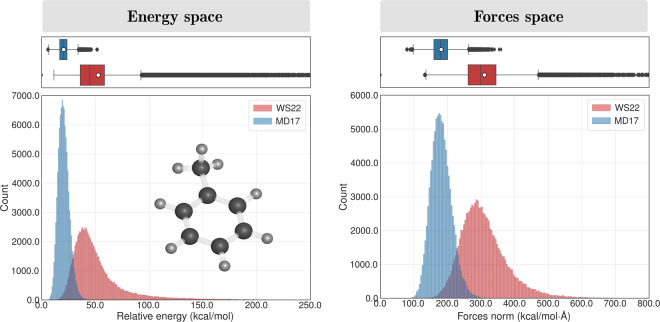
Statistical distribution of the potential energy (left panel) and Frobenius norm of forces matrices (right panel) for toluene in the WS22 and MD17 datasets. Energy values are relative to the minimum of each dataset.

A similar tendency is observed when comparing the data distribution in the atomic forces space. In this case, the histograms in Fig. 7 show that the norm (Frobenius) of the atomic forces matrices has a broad Gaussian shape centered on 298 kcal mol^−1^ Å^−1^ in the WS22 dataset, while in MD17, the data is narrowly distributed around 178 kcal mol^−1^ Å^−1^. As for the standard deviation, we found the values of 29 kcal mol^−1^ Å^−1^ for MD17 and 57 kcal mol^−1^ Å^−1^ for the WS22 dataset. It is worth stressing that, for the toluene dataset, we have considered only one equilibrium geometry (S_0_ minimum) as a reference to apply the Wigner sampling. Hence, the coverage of chemical properties in the other nine datasets composing the WS22 database should be even larger since our sampling strategy is applied to different conformational spaces.

Finally, the composed sampling strategy used to build the WS22 database gives rise to significant differences in configurational space covering and quantum properties distribution not observed in similar databases such as MD17. These differences lead to the natural question of how much they may affect the learning performance of typical MLP models. While out of the scope of this paper, one of us has conducted an independent ML study to answer this question. It was found that the WS22 dataset uncovered additional requirements for training and testing MLPs^56^. One of such requirements is that using independently constructed datasets is paramount for fair testing ML potentials, while currently, many tests are limited to only using the MD17 dataset.

## Usage Notes

As part of our database, we provide an interactive dashboard written in Python that is publicly available in the Streamlit cloud [ws22-database.streamlit.app]. The dashboard was designed to facilitate a preliminary data exploration and visualization of the main statistical features contained in the molecular datasets. Using this tool, the users can also visualize the molecular structures and download the geometries in the standard XYZ format.

## Data Availability

All the data annotations with the chemical properties were obtained from density functional theory calculations performed with the Gaussian 09 program^43^. The set of molecular geometries used as input for these calculations was generated using the Newton-X CS (version 2.2-B08) package^53^ for Wigner sampling together with a Python code for geometry interpolations [https://github.com/virtualzx-nad/geodesic-interpolate] as described in the Methods section. Both programs are open access. A custom Python script was written to extract the relevant information from the Gaussian 09 output files, and it is publicly available in the GitHub repository https://github.com/maxjr82/QCDP. Finally, the Python script to perform the dimension reduction of the molecular geometries with the PCA method is available to download from https://github.com/maxjr82/PCA-for-WS22.
